# Supplementary material for: Electrophysiological Screening to Assess Foot Drop Syndrome in Severe Acquired Brain Injury in Rehabilitative Settings
Source: Biomedicines. 2024 Apr 16;12(4):878. doi: 10.3390/biomedicines12040878 (PMC11048380; doi:10.3390/biomedicines12040878)
Supplement: Supplementary file 1 [file biomedicines-12-00878-s001.zip › biomedicines-2934513-supplementary.pdf]

**Table S 1 :** Demographical and clinical characteristics at admission in IRU and at follow-up in the CIPNM subgroups

| | CIM (n = 6) | CIP (n = 4) |
|---|---|---|
| Age (years) | 64 [41-76] | 54 [43-75] |
| Sex (F/M) | 2/4 | 4/0 |
| Traumatic aetiology | 1 | 1 |
| Vascular aetiology | 5 | 3 |
| Time post-onset (days) | 57 [26-70] | 37 [19-41] |
| Tracheostomy | 6 | 2 |
| PEG | 3 | 0 |
| Nasogastric tube | 3 | 2 |
| Decompressive Craniectomy | 1 | 0 |
| CRS-R at Admission | 10.5 [7-21] | 15 [3-23] |
| CRS-R at 2 months | 16 [4-23] | 20.5 [6-23] |
| LCF at Admission | 3 [2-5] | 4 [2-5] |
| LCF at 2 months | 4 [2-6] | 5.5 [2-7] |
| DRS at Admission | 22 [18-26] | 19 [17-26] |
| DRS at 2 months | 21.5 [16-24] | 16 [16-24] |
